# Comparison of the prevalence of malocclusion and oral habits between children with cerebral palsy and healthy children

**DOI:** 10.1186/s12903-023-03840-z

**Published:** 2024-01-11

**Authors:** Fuad Lutf Almotareb, Hassan Abdulwahab Al-Shamahy

**Affiliations:** 1 Orthodontics, Pedodontics and Prevention Department, Faculty of Dentistry, Sana’a University, Sana’a, Yemen; 2 Department of Basic Sciences, Faculty of Dentistry, Sana’a University, Sana’a, Republic of Yemen

**Keywords:** Cerebral palsy child, Prevalence, Malocclusion, Oral habits

## Abstract

**Background:**

Cerebral palsy (CP) represents for children an important problem of health and affects roughly 2 per 1000 live births and is the most common pediatric developmental motor disability. Therefore, the purpose of this study was to determine the prevalence, type and severity of malocclusion and oral habits in children with Cerebral Palsy (CP) and to compare them with a control group of healthy children in Sana’a city.

**Materials and methods:**

A prospective, case–control study was made of two groups, a cerebral palsy and a control group. The study population consisted of 60 children who had CP, and a control group of 60 matched children with no physical or mental disabilities. Data were collected using a questionnaire and assessment for malocclusion was done clinically. The patients were compared with equal number of age-matched controls. The inclusion criteria were individuals aged over 6 years; absence of previous orthodontic treatment; no missing permanent first molars.

**Results:**

Results showed an increased prevalence of malocclusion in children with cerebral palsy. Molar class II relationship was statistically higher in cerebral palsy children than healthy control (*P* = 0.001). Cerebral palsied children are likely to have a significantly increased protrusion of the anterior teeth (*P* < 0.001) when compared with normal children. Mouth breathing and Tongue thrust. Habits were significantly higher in the CP group (*p* = 0.0001) when compared with normal children.

**Conclusion:**

The prevalence of malocclusion was higher in children with Cerebral palsy than in normal children, and the present study concludes that in children with Cerebral Palsy, more oral Habits problems due to oral motor dysfunctions are common and problems of mouth breathing and Tongue thrust produce different malocclusion and poor oral hygiene complications in these children.

## Introduction

Cerebral palsy is a non- progressive disorder which is defined as abnormal of motion and posture and caused by damage to the motor control centers of the developing brain and can occur in the first year of life up to about age three, [1]. In CP, due to the abnormal muscle activity and forces, the orofacial dysfunctions could result in orofacial bone deformities. The muscles of the face and oral cavity play a role in facial growth and occlusal development [2]. As the tone and function of the orofacial muscles with CP can be abnormal, the facial growth and occlusion of these children may be outside normal limits. The amount of dentoalveolar skeletal deformation is related to the frequency, duration, direction, and intensity of the oral habits. Some of these habits are more commonly observed in some of these conditions, and it is possible that children with CP who have oral habit will have increased malocclusion. There are conflicting reports regarding the prevalence of malocclusion in children with CP. Some have found an increased prevalence of malocclusion [3–6], but others have found the prevalence of malocclusion to be within normal limits [7–10]. It has been suggested that malocclusion has a considerable impact on the lives of the children with CP and may be increased in the most severely brain damaged. The misalignment of teeth in cerebral palsy may be due to various problems related to oral functions, such abnormal movements of the tongue, lips and cheeks.

In a study of Greek children with disabilities, the highest rate of malocclusion was observed in children with cerebral palsy [11]. No specific oral pathology problems are associated with cerebral palsy children; the same diseases that affect the overall population can affect children with cerebral palsy, although with a higher prevalence or severity due to their weakened motor skills [12]. The orthodontic treatment of children with CP is difficult mainly by the lack of patient cooperation, due to their high sensitivity to physical contact and position at the dental chair [13].

In Yemen there are limited data available relating to dental health of patients with CP, thus, we conducted this study to observe the prevalence of malocclusion and associated oral Habits in cerebral palsy children in Yemen, and compared it with matched control normal children.

## Materials and methods

Case-control study design was carried out to compare the prevalence of malocclusion and oral habits in children with Cerebral Palsy (CP) with a control group of healthy children in Sana’a city, the capital of Yemen. The children in both groups in the study ranged from 6 to 13 years of age. Sample size estimation was based on information available from previous studies. The sample size was 120 children divided into two groups: 60 cerebral palsy (CP) patients and 60 healthy controls.

This sample size was calculated by considering the following:

Two sided confidence level = 95%., Case: control = 1:1 and Power of study = 80%.

Ratio of control exposure to malocclusion from previous study not less than = 7% [14].

Odd’s ratio of malocclusion in cases = 4.7 and Present of malocclusion in cases = 26% [15].

Therefore, 60 cases and 60 controls are needed to achieve statistically significant results.

The CP children were examined in their special education centers, while the control group were examined at the dental clinics of the College of Dentistry, Sanaa University. Consent for examining of the children was obtained from parent prior to oral examination of the children. Ethical approval was obtained from the Research Committee, Sanaa University in Yemen.

The presence of malocclusions and oral habits were examined. Dental examination were used to record the presence of Class I malocclusion, Class II malocclusion, Class III malocclusion as given by Angle, anterior open bite, anterior and posterior cross bite, and protrusion of the anterior teeth.

The presence of habits was investigated through a questionnaire answered by the caregiver followed by the clinical examination of the presence of tongue thrust, while presence of mouth breathing was diagnosed through mirror test and water retention test in the CP group and in the control group.

The inclusion criteria were children previously diagnosed with CP, aged over 6 years; absence of previous orthodontic treatment; no missing permanent first molars. Children who were not able to cooperate due to their severe intellectual disability were excluded from the study.

Data were statistical analyzed using (SPSS Statistics for windows version 28.0; IBM Corp).

## Results

### Malocclusion

The children included in the study were within the age range of 6 and 13 years. Sixty Children with cerebral palsy were compared with the 60 healthy children. Children with cerebral palsy have shown significantly higher proportion of malocclusion compared with healthy children. The prevalence of malocclusion revealed amongst children with cerebral palsy is presented in Table 1. Out of 60 children with cerebral palsy, thirty (50%) of them had Class I malocclusion while 28(46,7%) had Class II malocclusion and 2(3,3%) had class III. Out of the controls children, 43(71,7%) had Class I malocclusion while 12 (20%) had Class II malocclusion and 5 (8,3%) had class III. The following malocclusions were diagnosed: anterior cross bite in 2 (3,3%) cases, protrusion of the anterior teeth was diagnosed in 12 (20%) cases and anterior open bite was diagnosed in 20 (33.3%) cases and Deep bite was 10 (16.7%) as shown in Table 1. The anterior open bite with protrusion of the anterior teeth, together with abnormal muscle movement and posture problems, are responsible for much of the trauma to anterior teeth. After data entry, the following comparisons were then made for each of the variables measured: the healthy children was compared with children with CP. The children with cerebral palsy demonstrated a statistically significant Class II malocclusion (< 0.001) with evidence for an association between CP group and Class II malocclusion (odds ratio [OR] 3,5% confidence interval [CI] 1.6 to 7.9), and no significant difference in the Angle’s class I malocclusion and Angle’s class III malocclusion was detected between the two groups. Significant difference was noted between cases and controls for protrusion of the anterior teeth (< 0.0001). There were no significant differences between any of the comparisons in the following variables: anterior open bite, Deep bite, anterior cross bite and posterior cross bite.

**Table 1:** Prevalence of different types of malocclusion in the CP group and in the control group

Factors	Cases n = 60		Controls n = 60		*OR*	*CI*	*X* ^*2*^	*P*
Malocclusion	No	%	No	%				
Class I malocclusion	30	50	43	71.7	0.3	0.18–0.8	5,9	0.01
Class II malocclusion	28	46.7	12	20	3.5	1.6–7.9	9.6	0.001
Class III malocclusion	2	3.3	5	8.3	0.37	0.07-2	1.3	0.24
Post. Cross bite	4	6.7	5	8.3	0.78	0.2-3	0.12	0.72
Open bite	20	33.3	20	33.3	1.0	0.4–2.1	9,9	1.0
Deep bit	10	16.7	15	25	0.6	0.24–1.4	1.2	0.26
Ant. Cross bite	2	3.3	8	13.3	0.22	0.04–1.1	3.9	0.04
protrusion of the anterior teeth	12	20	0	0	Undefined		13.2	< 0.0001

### Oral habits

In our study, 11.7% of the healthy children group presented mouth breathing compared to 60% of the Children with cerebral palsy, while the majority of the Children with cerebral palsy group presented Tongue thrust 36.7% compared to 5% of the healthy children group. Mouth breathing and Tongue thrust Habits were significantly higher in the Children with cerebral palsy group (*p* = 0.0001). With increasing association between Children with cerebral palsy group and mouth breathing, Tongue thrust Habits (odds ratio [OR] 3.5% confidence interval [CI] 1.6 to 7.9).

For Thumb sucking Habits no significant difference was observed between cases and controls.

The habits are compared in Table 2.

**Table 2:** Prevalence and association of oral habits in the CP group and in the control group

Oral habits	Cases n = 60		Controls n = 60		*OR*	*CI*	*X* ^*2*^	*P*
	No	%	No	%				
Mouth breathing	36	60	7	11.7	11.4	4.4–29	30.4	< 0.0001
Thumb sucking	12	20	10	16.7	1.2	0.49–3.1	0.22	0.65
Tongue thrust	22	36.7	3	5	11	3.1–39.3	18.2	< 0.0001

## Discussion

The purpose of this study was to determine the prevalence, type, and severity of malocclusion and oral habits in children with cerebral palsy (CP) and to compare them with a control group of healthy children in Sanaa City. Many authors show data showing that children with CP have a larger frequency of sagittal, vertical and transversal malocclusions because of the misbalance between perioral and intraoral muscles [16, 17]. The present study adds to the evidence that there is an increased prevalence of malocclusion in children with CP. We found that there was significant difference between children with cerebral palsy and control group of healthy children for class II malocclusion. In this study, we found 46.7% of CP children group had Class II malocclusion, which is significantly higher as compared to normal child; these results are Consistent with the findings of other studies [18, 19].

The study conducted by Sinha N et al., Chandna et al., and Miamoto CB et al., reported 58%, 60%, and 46.8% class II malocclusion. In a study, it was observed that the prevalence of class II malocclusion is significantly higher in CP children as compared to normal child [20–22]. In agreement with other authors, showed class III to be the least prevalent malocclusion in both groups of individuals, while class I was found to be the most prevalent presentation among the control group [22–24]. The results of the study showed that there was an increased prevalence of anterior teeth protrusion in children with CP when compared to the normal children. This high prevalence was in agreement with the findings in other studies [22, 24]. In the present study, the prevalence of mouth breathing found in the CP group (60%), was greater than that found in the control group (11, 7%) and this difference was statistically significant (*P* < 0.0001), these results are Consistent with the findings of the study in Brazil, showed that the prevalence of mouth breathing in the sample was 56.8% of the sample studied [25]. A similar study conducted by Savian et al., which showed the prevalence of mouth breathing was 44%, these results are Consistent with the findings [26]. In this study, children with Cerebral Palsy had remarkably high frequencies of tongue thrust (36.7%) when compared to control group of healthy children. These findings were confirmed by another study conducted in Sharjah city [27]. This result is lower than that reported by other authors in India, in this latter study, 79% of children with Cerebral Palsy had tongue thrust [28]. Future studies should account to describe the association between abnormal oral function and orofacial bone deformities in children with Cerebral Palsy. This study will represent the best available data, which informing our understanding of the epidemiology of children with Cerebral Palsy in Yemen and highlight any knowledge gaps for researchers to bridge these existing gaps.

## Conclusions

In conclusion, the prevalence of malocclusion among Yemeni children with CP was high and associated with behavioral tongue thrusting and mouth breathing compared to normal children. Children with CP had significantly higher Class II molar relationships. In addition, they demonstrated significantly increased protrusion of the front teeth compared to the controls. Children with CP had significantly more mouth breathing and tongue thrusting than healthy children. The general health of children with cerebral palsy can be improved with early intervention, which can minimize the extent of disabilities and prevent negative health outcomes.

## Limitations

The results interpreted from the included studies are limited mainly by the small sample sizes.

## Future recommendations

Improve data collection systems, through building a strong knowledge and evidence database and Improve access to health services for Children with intellectual disability.

## Data Availability

The data that support the findings of this study are available from the corresponding author upon reasonable request.

## Declarations

### Declaration

I hereby declare that this research is my own work and effort and that it has not been submitted anywhere for any award.
